# Quantifying germination cardinal temperatures of ten forage legumes using non-linear regression models

**DOI:** 10.3389/fpls.2026.1754589

**Published:** 2026-02-23

**Authors:** Mengyao Shi, Yumeng Hu, Ruoxi Jia, Xinli Zhao, Jiahui Tan, Haoyang Teng, Yiran Han, Yihao Gai, Zitong Lei, Yichen Yuan, Shangzhi Zhong, Juan Sun, Qibo Tao

**Affiliations:** 1 Shandong Key Laboratory for Germplasm Innovation of Saline-alkaline Tolerant Grasses and Trees, Qingdao Key Laboratory of Specialty Plant Germplasm Innovation and Utilization in Saline Soils of Coastal Beach, College of Grassland Science, Qingdao Agricultural University, Qingdao, China; 2 College of Animal Science and Technology, Qingdao Agricultural University, Qingdao, China

**Keywords:** cardinal temperature, forage legumes, germination rate, non-linear regression models, thermal range

## Abstract

**Introduction:**

Forage legumes play a pivotal role in livestock production, environmental protection, sustainable cropping systems, and various industrial applications. Understanding the germination thermal requirements of forage legumes is necessary for optimizing their sowing and production. The response of germination rate to temperature can be described using non-linear regression models.

**Methods:**

In this study, ten constant temperatures (from 0 to 45°C with 5°C interval) were evaluated, and two non-linear regression models (intersected-lines and quadratic polynomial) were applied to quantify cardinal temperatures and thermal ranges for ten important forage legumes.

**Results and discussion:**

Both germination percentage and germination speed were low in hot and cold temperatures outside the range of 15-25°C for these species. Notably, significant inter-species variation in thermal requirements was identified. Alfalfa (*Medicago sativa*), yellow medick (*Medicago falcata*), and erect milkvetch (*Astragalus adsurgens*) demonstrated high thermal plasticity, characterized by low minimum (base) temperature (*T_b_*), high maximum temperature (*T_m_*), and wide thermal ranges. Conversely, sweet clover (*Melilotus officinalis*) exhibited a preference for cooler thermal regimes, with the lowest optimum temperature (*T_o_*) and *T_m_* thresholds. Niuzhizi (*Lespedeza potaninii*) and white clover (*Trifolium repens*) were identified as thermophilic species, requiring higher temperatures for optimal germination, as evidenced by their higher *T_o_* and *T_m_* thresholds, whereas species of the genus *Vicia* and red clover (*Trifolium pratense*) were best adapted to moderate thermal environments. Furthermore, germination speed-related parameters were more sensitive to temperature fluctuations than germination percentage. Among the tested models, the intersected-lines model showed superior performance for predicting the cardinal temperatures of the ten forage legumes, as indicated by a lower root mean square of error (RMSE) and a higher coefficient of determination (*R²*). No statistically significant relationships between cardinal temperatures and germination parameters were observed. These findings provide a scientific basis for improving sowing practices and regional species selection, which is vital for forage legume cultivation and production. The identification of cardinal temperatures is also crucial for the development of plant growth and biomass prediction models that simulate growth and estimate yield under current and future climate scenarios.

## 1 Introduction

Over the past three decades, considerable economic and social development, coupled with rising living standards, has significantly shifted dietary habits in China and globally, marked by increased consumption of animal-sourced foods, such as meat, milk, and eggs (Notenbaert et al., 2021; Sun et al., 2024). Concurrently, rapid world population growth has further escalated food consumption, and animal-based food consumption in particular (Qaim et al., 2024). Nowadays, livestock products, including meat, milk, and eggs, contribute 15% and 31% of the global per capita calorie and protein supply, respectively (Godde et al., 2021), underscoring a growing global demand for high-quality forage production as livestock feed. There is likely to be an increasing emphasis on the role of forage legumes in producing high-quality meat and milk, combined with a reduction in environmental footprint (Boelt et al., 2015). Forage legumes are of vital importance to animal husbandry development, sustainable animal-based food production, and environmental protection (Phelan et al., 2015). Due to their high protein productivity, nitrogen fixation capacity, and adaptability to diverse marginal lands (Tao et al., 2022; Sollenberger and Kohmann, 2024), forage legumes perform well in livestock production and environmentally sustainable cropping systems (Stagnari et al., 2017). Under global climate change scenarios, incorporating forage legumes into grassland and agricultural systems is a highly recommended strategy to enhance crop diversity, thereby mitigating climate change (Jensen et al., 2012). However, poor establishment is a persistent problem associated with forage legumes, especially for small-seeded forage legume species (Wang et al., 2004). The effects of unfavorable weather conditions are arguably more critical during germination and early seedling growth stages than at any other stage of vegetative and reproductive growth. Seedling emergence and successful establishment are especially difficult to achieve for spring- or autumn-planted forage legumes due to frequently occurring erratic weather conditions, temperature fluctuations in particular, under global climate change (Gesch et al., 2023).

Seed germination and emergence are among the most important stages in the establishment of a plant species and the most sensitive stages in the plant life cycle to environmental conditions (Zhu et al., 2014). Rapid and uniform seed germination and emergence are of great importance for successful stand establishment, which ultimately improves forage yield and quality (Tao et al., 2024). Seed germination is a complex physiological process that is affected not only by seed properties but also by various environmental factors (Bakhshandeh et al., 2020). Among these environmental factors, temperature is one of the most crucial limiting factors for seed germination, dormancy, and plant establishment (Zhang et al., 2022; Nikolić et al., 2023). Prolonged exposure to high temperatures can induce secondary dormancy in seeds (Martel et al., 2018). As a key environmental driver, temperature regulates enzyme activity and promotes or inhibits the synthesis of hormones that influence seed germination (Sharma et al., 2022). Therefore, temperature determines plant metabolism and developmental rates and has significant effects on the onset, percentage, and rate of germination (Chen et al., 2021). Appropriate temperature conditions for seed germination and seedling growth are critical factors in determining the optimum sowing date of a species (Russo et al., 2010). Moreover, the thermal requirements for germination and seedling growth are also pivotal indices for determining the suitability of a crop for cultivation in new areas (Adam et al., 2007). Thus, knowledge of seed germination responses to temperature is required not only for understanding the ecological adaptation of species but also for formulating effective strategies for sowing and restoration (Chen et al., 2021).

The impacts of temperature on germination can be expressed as three “cardinal temperatures” (Alvarado and Bradford, 2002; Parmoon et al., 2015). In general, the minimum, optimum, and maximum temperatures are known as cardinal temperatures and are used to characterize seed germination responses to temperature (Bakhshandeh et al., 2013), defining the thermal range within which germination can occur (Al-Ahmadi and Kafi, 2007). The minimum (or base, *T_b_*) and maximum (or ceiling, *T_m_*) temperatures are those below and above which germination does not occur, respectively, while germination is most rapid at the optimum temperature (*T_o_*) (Eberle et al., 2014). In most plant species, cardinal temperatures for germination are roughly similar to those required for vegetative growth stages (Solouki et al., 2022). Nevertheless, some studies have reported that, in certain species, cardinal temperatures for germination differ from those governing root or stem growth (Adam et al., 2007).

Non-linear regression models have been successfully applied to describe germination cardinal temperatures for staple crops (Ali et al., 1994), oil crops (Bidgoly et al., 2018), medicinal plants (Solouki et al., 2022), weeds (Ma et al., 2021), and many other plant species. For instance, the intersected-lines model consists of two linear regressions: in the first line, germination rate increases up to the optimum temperature threshold, whereas in the second line, germination rate decreases in response to further temperature increases. Base and maximum temperatures are derived from the interception of each regression line with the abscissa (x-axis). In addition, the optimum temperature is identified as the intersection point of the two linear regression lines (Fallahi et al., 2015). Previously, Zhang et al. (2020) explored germination cardinal temperatures of seven *Stipa* species from different habitats using the intersected-lines model and reported that *Stipa* species from cool habitats had higher germination cardinal temperatures than those from warm habitats. In addition, other non-linear regression models, such as the quadratic polynomial, have also been widely used to determine germination cardinal temperatures (Solouki et al., 2022). In a previous study on the germination response of *Alyssum linifolium* to temperature, *T_b_*, *T_o_*, and *T_m_* values calculated using a quadratic polynomial model were 3.3, 19.1, and 35.0 °C, respectively (Mobli et al., 2018).

Several studies have reported the effects of temperature on germination traits of forage legumes (Brar et al., 1991). Butler et al. (2014) determined the influence of temperature on germination percentage in seven annual warm-season and 11 annual cool-season forage legumes. Baxter et al. (2019) reported that the germination of crimson clover (*Trifolium incarnatum*), balansa clover (*Trifolium michelianum*), red clover (*Trifolium pratense*), and white clover (*Trifolium repens*) maintained values above 80% across a temperature range of 4.9–28.2 °C on average. Ibañez and Passera (1997) reported that the optimum temperature for albaida (*Anthyllis cytisoides*) germination was 20 °C. For grain legume crops, the *T_b_* values for pea (*Pisum sativum*) and bean (*Phaseolus vulgaris*) were −1.1 and 5.1–9.6 °C, respectively (Raveneau et al., 2011). Nevertheless, to the best of our knowledge, a comprehensive and systematic evaluation of germination cardinal temperatures and thermal ranges for important forage legumes remains scarce. Previously, Dürr et al. (2015) developed a global seed trait database encompassing germination trait data for 243 species; however, within the forage legume category, only *T_b_* values for alfalfa (*Medicago sativa*), red clover, and white clover were reported, with values of 2.9, 3.9, and 4.2 °C, respectively. This highlights the fragmentary nature of existing thermal trait records for this economically important group. In addition, Sakanoue (2010) employed the reciprocal of the time taken for 50% germination (1/T_50_) to calculate *T_b_* for these three forage legumes, which differs from the approach used in the present study, where germination rate (GR) was applied as the dependent variable. Given the crucial roles of forage legumes in livestock production, sustainable cropping systems, and environmental protection, confirmatory testing is important for accurately quantifying germination thermal requirements and cardinal temperatures to select suitable cultivation regions and sowing periods. Moreover, to address current challenges during forage legume stand establishment, an improved understanding of germination biology—particularly germination cardinal temperatures and thermal ranges—is required. Quantifying *T_b_*, *T_o_*, and *T_m_* provides a physiological framework for predicting safe sowing windows, thereby minimizing the risk of seedling mortality caused by frost or heat stress during emergence and early development (Parmoon et al., 2015). Furthermore, characterizing species-specific thermal niches enables more accurate matching between seed physiology and regional environmental conditions (Donohue et al., 2010). By integrating these biological parameters into cropping models, researchers and producers can better mitigate the impacts of erratic weather, ensuring more reliable establishment and long-term sustainability of cropping systems incorporating forage legumes (Basche et al., 2016).

The present research was conducted using seeds of ten important forage legumes: (i) to evaluate the impacts of different temperatures on germination characteristics, (ii) to calculate germination thermal requirements and cardinal temperatures using two commonly employed non-linear regression models, and (iii) to compare the performance of the two models in calculating cardinal temperatures. This extensive study should enhance our understanding of ecological adaptation in the tested forage legume species and offer valuable insights to researchers, agronomists, farmers, and government program directors for optimizing sowing and agronomic management practices, as well as for improving establishment and production efficiency. The data collected in this study also represent a valuable enrichment of global seed trait databases, enabling plant establishment to be better incorporated into modeling and simulation studies of forage legume biogeographical boundaries in response to shifting land use and climate.

## 2 Materials and methods

### 2.1 Seeds of ten selected forage legumes used in this experiment

In the present experiment, ten important forage legume species were selected to evaluate the impacts of temperature on germination performance and to calculate their germination cardinal temperatures. These species included alfalfa, yellow medick (*Medicago falcata*), common vetch (*Vicia sativa*), hairy vetch (*Vicia villosa*), red clover, white clover, erect milkvetch (*Astragalus adsurgens*), Chinese milkvetch (*Astragalus sinicus*), Niuzhizi (*Lespedeza potaninii*), and sweet clover (*Melilotus officinalis*). These species were chosen due to their widespread utilization in China and globally, where they play important roles in forage production, livestock development, nitrogen fixation, soil improvement, sustainable cropping systems, water and soil conservation, urban landscaping, ecological restoration, industrial applications, and other sectors. Seeds of the ten species used in this study were obtained from commercially available sources. Additional details, including thousand seed weight (TSW), seed moisture content (SMC), standard germination (SG), and hard seed (HS), are summarized in **Table 1**. Seed moisture content was calculated on the basis of seed fresh weight.

**Table 1:** Information of ten selected forage legume seeds applied in this experiment.

Species	Common name	Cultivar or variety	TSW (g)	SMC (%)	SG (%)	HS (%)
*Medicago sativa*	Alfalfa	Zhongmu No.3	2.141	9.12	93.0	1.0
*Medicago falcata*	Yellow medick	Normal	1.474	10.41	93.0	3.5
*Vicia sativa*	Common vetch	Lanjian No.1	54.274	8.95	97.5	0.0
*Vicia villosa*	Hairy vetch	Normal	36.443	9.31	96.5	0.0
*Trifolium pratense*	Red clover	Honglong	1.751	8.31	84.5	5.5
*Trifolium repens*	White clover	Haifa	0.552	8.69	82.5	4.0
*Astragalus adsurgens*	Erect milkvetch	Normal	1.647	7.03	87.5	4.0
*Astragalus sinicus*	Chinese milkvetch	Yujiangdaye	3.494	9.10	92.5	2.0
*Lespedeza potaninii*	Niuzhizi	Tenggeli	2.268	10.27	72.5	9.0
*Melilotus officinalis*	Sweet clover	Normal	2.256	10.05	95.5	2.0

‘Normal’ in the cultivar or variety name reflects that there is no specific cultivar or variety for this species. TSW, thousand seed weight; SMC, seed moisture content; SG, standard germination; HS, hard seed. The SMC was calculated on basis of seed fresh weight.

### 2.2 Experimental design

To determine the effects of temperature on seed germination, an experiment was conducted in the Forage Seed Laboratory of Qingdao Agricultural University, China (36°19′N, 120°23′E), from November to December 2024. Ten constant temperature levels (i.e., 0, 5, 10, 15, 20, 25, 30, 35, 40, and 45 °C) were applied using a completely randomized design with four replications for each species. Prior to the germination assays, seeds of the ten forage legumes were surface-sterilized in 1% sodium hypochlorite (NaClO) for 10 min and then rinsed several times with distilled water. For each replication, 50 seeds were sown in 120 mm × 120 mm Petri dishes on top of two layers of filter paper moistened with 10 mL distilled water. The Petri dishes were then sealed with parafilm to minimize moisture loss. Germination tests were performed in growth chambers (GXZ-380, Jiangnan, Ningbo, China) under the respective constant temperatures and set to a 12-h alternating light/dark cycle. Seeds were considered germinated when the radicle attained a length of 2 mm (Kamkar et al., 2012), and germinated seeds were counted daily for 28 days.

### 2.3 Calculation of germination parameters

The following seed germination parameters were calculated to describe the response of germination to temperature treatments more comprehensively.

The time to start germination (TSG) was recorded during the germination experiment (Farooq et al., 2005).

At the end of the germination period, germination percentage (GP) was determined as the total number of seeds germinated divided by the total number of seeds (50) in each replication. The formula provided by Xiong et al. (2018) was applied to calculate the germination index (GI):


$$\text{GI}=\frac{n_{1}}{1}+\frac{n_{2}}{2}+\cdot \cdot \cdot \cdot \cdot \cdot +\frac{n_{i}}{i}$$

where *n_1_*, *n_2_*,……, *n_i_* is the number of seeds newly germinated or emerged on day *1*, *2*,……, *i*.

The GR was calculated with the formula described by Fallahi et al. (2017):


$$GR\;\left(day^{-1}\right)\;=\;\sum_{i=1}^{n}\frac{Si}{Di}$$

where *Si* is the daily seed germination, *Di* is the number of days to *n* computation and *n* is the number of days to computation.

The T_50_, which also gave an estimate of germination speed, was determined according to the following formula of Yang et al. (2012):


$${\text{T}}_{50}\;\left(\text{days}\right)=t_{i}+\frac{\left(\frac{N}{2}-n_{i}\right)\left(t_{j}-t_{i}\right)}{\left(n_{j}-n_{i}\right)}$$

where *N* is the final number of germination and *n_i_* and *n_j_* the cumulative number of seeds germinated by adjacent counts at time *t_i_* and *t_j_* when *n_i_*< *N/2*< *n_j_*.

The mean germination time (MGT) was calculated with the formula described by Tao et al. (2018):


$$\text{MGT }\left(\text{days}\right)=\frac{\text{Σ}Dn}{\text{Σ}n}$$

where *n* represents the number of seeds newly germinated on day *D*, and *D* is the number of days counted since the initiation of the germination experiment.

### 2.4 Germination cardinal temperatures

To calculate the cardinal temperatures of the ten selected forage legumes, the relationship between germination rate (GR) and temperature was analyzed, with temperature considered the independent variable (x-axis) and GR the dependent variable (y-axis) (Solouki et al., 2022). In the present research, two commonly used non-linear regression models—namely, the intersected-lines and quadratic polynomial models—were employed (Fallahi et al., 2015).

The intersected-lines model is expressed as:


$$T_{1}\;=\;b\left(T\;–\;T_{b}\right)\;if\;T\;\leq \;T_{o}$$

$$T_{2}\;=\;c\left(T_{m}\;–\;T\right)\;if\;T\;\geq \;T_{o}$$

where *T* is temperature treatment; *T_b_*, *T_o_*, and *T_m_* are base, optimum, and maximum temperatures, respectively; *b* and *c* are model parameters.

The quadratic polynomial model is as follows:


$$f\;\left(T\right)\;=\;a\;+\;bT\;+\;cT^{2}$$

$$T_{o}\;=\;b\;+\;2cT$$

where *f(T)* is GR; *T* is temperature treatment; *a*, *b*, and *c* are model parameters.

The performance of each model was evaluated using the coefficient of determination (*R²*) and the root mean square of error (RMSE). Lower RMSE values and *R²* values closer to 1 indicate better model performance (Fallahi et al., 2017).


$$RMSE\;=\;\sqrt{\frac{\sum_{i\;=\;1}^{n}{\left(O_{i}\;-\;S_{i}\right)}^{2}}{n}}$$

In this context, RMSE represents the root mean square of error, *n* is the number of samples, *O*_i_ denotes the observed values, and *S*_i_ denotes the predicted values.

Additionally, the temperature difference between *T_b_* and *T_m_* was defined as the thermal range. This index represents the germination ecological range of a plant species (Solouki et al., 2022).


$$Thermal\;range\;\left({}^{\circ}C\right)\;=\;T_{m}\;–\;T_{b}$$

### 2.5 Statistical analysis

All data analyses were performed using GenStat for Windows, 18th Edition (VSN International Ltd., Hemel Hempstead, UK). One-way analysis of variance (ANOVA) was conducted to assess the effects of temperature on germination percentage (GP) and related germination parameters within each species. Proportional data were arcsine-transformed prior to statistical analysis to meet the assumption of normality (Tang et al., 2024), whereas non-transformed data are presented in all tables and figures. All values reported are means of four replications. Significant differences among temperature treatments were determined using Duncan’s multiple range test at the *P* < 0.05 probability level. Pearson correlation analysis was conducted to examine relationships among germination cardinal temperatures, thermal range, and germination parameters across the ten forage legume species, using mean germination parameter values averaged across all temperature treatments within each species.

## 3 Results

### 3.1 Effects of different temperatures on GP of ten forage legumes

**Figure 1:**
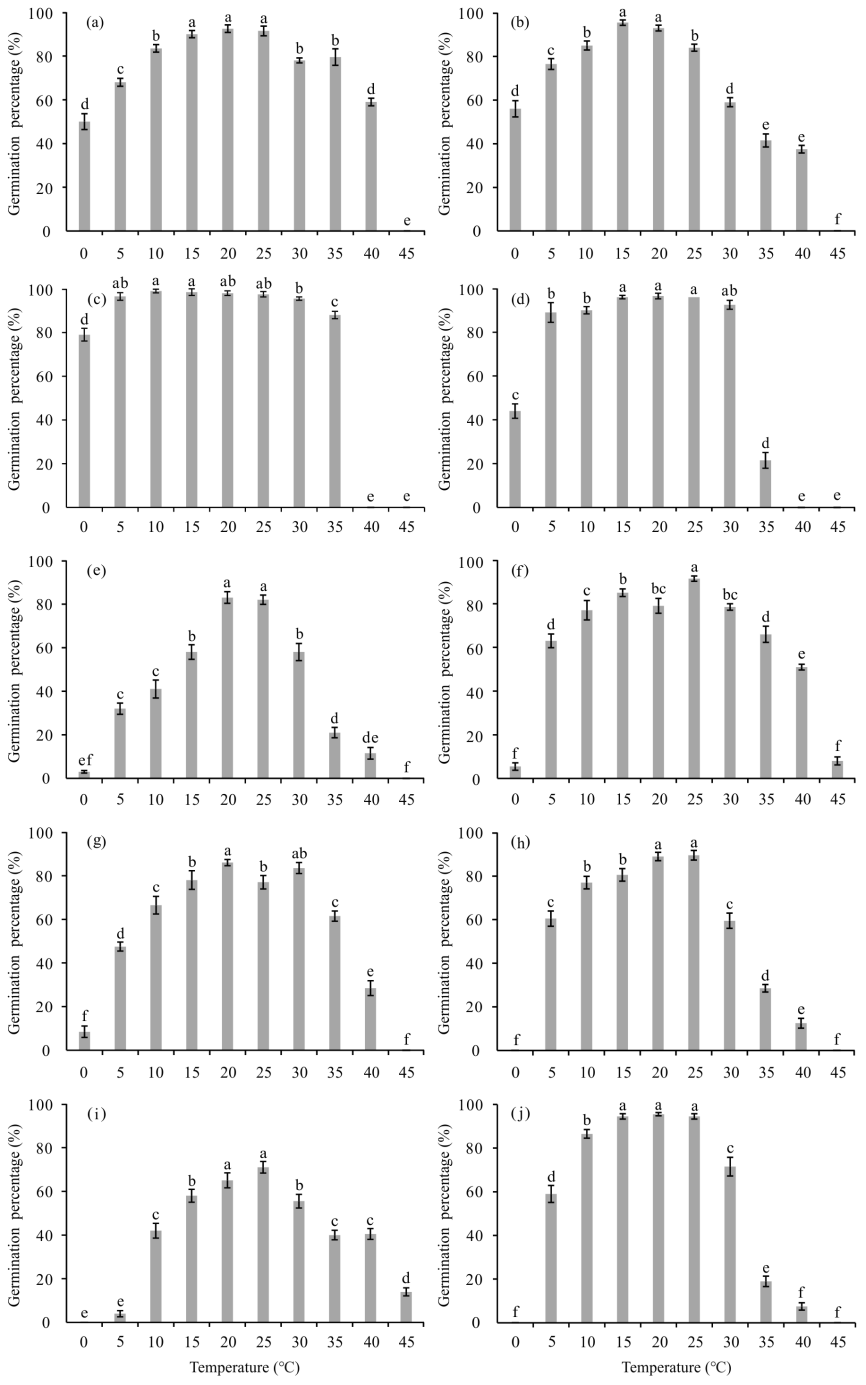
Germination percentage (GP) of ten forage legumes as affected by temperature treatments. Different lowercase letters within a species indicate significant differences among different temperature treatments at the *P* < 0.05 probability level according to Duncan’s multiple range test. **(a)** alfalfa (*Medicago sativa*); **(b)** Yellow medick (*Medicago falcata*); **(c)** common vetch (*Vicia sativa*); **(d)** hairy vetch (*Vicia villosa*); **(e)** red clover (*Trifolium pratense*); **(f)** white clover (*Trifolium repens*); **(g)** erect milkvetch (*Astragalus adsurgens*); **(h)** Chinese milkvetch (*Astragalus sinicus*); **(i)** Niuzhizi (*Lespedeza potaninii*); **(j)**: sweet clover (*Melilotus officinalis*).

Analysis of variance revealed that GP of the ten forage legumes was significantly affected by temperature (*P* < 0.001) (**Table 2**). In general, GP increased as germination temperature approached the optimum and subsequently declined with further temperature increases (**Figure 1**). Most tested species exhibited optimal GP within the 15–25°C range. For five species—alfalfa, hairy vetch, red clover, erect milkvetch, and sweet clover—the highest GP values were observed at 20°C. The highest GP for white clover, Chinese milkvetch, and Niuzhizi occurred at 25°C, whereas common vetch and yellow medick achieved their highest GP at 10 and 15°C, respectively (**Figure 1**). All tested forage legumes were capable of germination at 0°C, except for Chinese milkvetch, Niuzhizi, and sweet clover, which showed no germination at this temperature. Only white clover and Niuzhizi maintained limited germination at 45°C (8.0% and 14.0%, respectively), whereas the remaining species failed to germinate (**Figure 1**).

**Table 2:** Analysis of variance for temperature effects on seed germination percentage and related germination parameters of ten forage legumes.

Species	Germination indicators	*df*	Mean square	*F*	*P*
Alfalfa (*Medicago sativa*)	GP	9	3166.044	164.898	< 0.001
	TSG	8	24.944	898.000	< 0.001
	GI	9	794.138	214.045	< 0.001
	GR	9	58.457	18.723	< 0.001
	T_50_	8	30.558	2352.624	< 0.001
	MGT	8	34.795	636.123	< 0.001
Yellow medick (*Medicago falcata*)	GP	9	3648.711	195.467	< 0.001
	TSG	8	31.028	124.111	< 0.001
	GI	9	266.508	188.527	< 0.001
	GR	9	12.831	151.884	< 0.001
	T_50_	8	37.888	80.983	< 0.001
	MGT	8	38.976	76.016	< 0.001
Common vetch (*Vicia sativa*)	GP	9	6435.822	1026.993	< 0.001
	TSG	7	25.424	221.883	< 0.001
	GI	9	592.253	423.756	< 0.001
	GR	9	108.354	45.733	< 0.001
	T_50_	7	36.433	633.134	< 0.001
	MGT	7	53.722	483.136	< 0.001
Hairy vetch (*Vicia villosa*)	GP	9	6930.989	326.420	< 0.001
	TSG	7	38.567	176.306	< 0.001
	GI	9	406.182	496.951	< 0.001
	GR	9	60.856	24.178	< 0.001
	T_50_	7	68.626	343.013	< 0.001
	MGT	7	81.245	534.439	< 0.001
Red clover (*Trifolium pratense*)	GP	9	3758.767	125.432	< 0.001
	TSG	8	79.819	82.889	< 0.001
	GI	9	294.127	157.283	< 0.001
	GR	9	22.687	37.793	< 0.001
	T_50_	8	119.389	70.184	< 0.001
	MGT	8	108.900	69.511	< 0.001
White clover (*Trifolium repens*)	GP	9	3736.767	135.227	< 0.001
	TSG	9	52.278	74.683	< 0.001
	GI	9	536.039	123.017	< 0.001
	GR	9	37.939	43.538	< 0.001
	T_50_	9	82.391	155.114	< 0.001
	MGT	9	90.605	261.773	< 0.001
Erect milkvetch (*Astragalus adsurgens*)	GP	9	3950.933	122.700	< 0.001
	TSG	8	35.278	63.500	< 0.001
	GI	9	159.748	117.671	< 0.001
	GR	9	22.350	27.550	< 0.001
	T_50_	8	68.492	193.589	< 0.001
	MGT	8	64.729	158.958	< 0.001
Chinese milkvetch (*Astragalus sinicus*)	GP	9	5248.267	228.186	< 0.001
	TSG	7	50.496	210.764	< 0.001
	GI	9	127.594	133.190	< 0.001
	GR	9	10.039	69.614	< 0.001
	T_50_	7	70.936	45.998	< 0.001
	MGT	7	72.233	59.014	< 0.001
Niuzhizi (*Lespedeza potaninii*)	GP	9	2540.667	98.475	< 0.001
	TSG	8	128.882	49.359	< 0.001
	GI	9	236.980	207.326	< 0.001
	GR	9	27.969	36.463	< 0.001
	T_50_	8	157.749	94.200	< 0.001
	MGT	8	153.778	83.978	< 0.001
Sweet clover (*Melilotus officinalis*)	GP	9	6931.156	386.496	< 0.001
	TSG	7	14.786	44.357	< 0.001
	GI	9	174.711	97.430	< 0.001
	GR	9	11.812	173.894	< 0.001
	T_50_	7	35.114	22.839	< 0.001
	MGT	7	35.411	39.753	< 0.001

*df*, degree of freedom; GP, germination percentage; TSG, time to start germination; GI, germination index; GR, germination rate; T_50_, time taken for 50% germination; MGT, mean germination time.

### 3.2 Effects of different temperatures on related germination parameters of ten forage legumes

The ANOVA results showed that temperature significantly affected all measured germination parameters at the *P* < 0.001 level (**Table 2**). Germination timing and speed parameters exhibited distinct response patterns across the temperature gradient (**Table 3**). Germination duration parameters, including TSG, T_50_, and MGT, followed a U-shaped trend, decreasing sharply as temperature increased from 0 °C toward the optimum range—reflecting thermal acceleration of metabolic processes—and increasing again beyond the optimum. For example, in alfalfa, T_50_ decreased from 9.01 days at 0 °C to 0.71 days at 25 °C and then increased to 1.61 days at 40 °C, with no germination occurring at 45 °C (**Table 3**). In addition, the first seed of all tested species germinated on day 1 at optimum temperature ranges, except for erect milkvetch, which exhibited a delayed onset of germination compared with the other species.

**Table 3:** Effects of different temperature treatments on germination traits of ten forage legumes.

Species	Temperature (°C)	TSG (days)	GI	GR (day^-1^)	T_50_ (days)	MGT (days)
Alfalfa (*Medicago sativa*)	0	8.25a	2.54g	1.23de	9.01a	10.29a
	5	4.00b	6.11f	2.90d	4.87b	5.94b
	10	1.00c	15.47e	5.79c	2.42c	3.11c
	15	1.00c	24.48d	8.25bc	1.52d	2.25d
	20	1.00c	36.43a	8.85b	0.79f	1.73e
	25	1.00c	38.39a	11.74a	0.71f	1.45e
	30	1.00c	32.29b	9.83ab	0.73f	1.46e
	35	1.00c	28.28c	7.61bc	1.05e	1.74e
	40	1.00c	15.41e	5.75c	1.61d	2.34d
	45	–	0.00g	0.00e	–	–
Yellow medick (*Medicago falcata*)	0	7.25a	2.86e	1.56cd	8.73b	10.61ab
	5	3.25c	5.65d	3.07b	7.46cd	7.94c
	10	1.00d	16.45c	5.17a	2.69e	3.60d
	15	1.00d	21.58a	5.50a	2.60e	3.92d
	20	1.00d	19.23b	3.36b	3.04e	4.24d
	25	1.25d	15.04c	3.44b	3.18e	4.29d
	30	2.75c	4.73d	1.77c	6.96d	8.23c
	35	6.25b	2.36e	1.30de	8.36bc	9.67b
	40	7.50a	1.74e	1.12e	10.53a	11.41a
	45	–	0.00f	0.00f	–	–
Common vetch (*Vicia sativa*)	0	7.25a	3.72f	1.74cd	9.43a	11.89a
	5	6.50b	6.15e	2.62bc	6.89b	8.37b
	10	2.50c	13.48d	3.72bc	3.08c	4.14c
	15	2.00d	22.77c	10.39a	1.62d	2.26d
	20	1.00e	23.09c	12.68a	1.66d	2.30d
	25	1.00e	29.43b	12.58a	1.40de	1.90de
	30	1.00e	33.43a	11.34a	1.12e	1.73e
	35	2.00d	13.06d	4.79b	3.40c	4.33c
	40	–	0.00g	0.00d	–	–
	45	–	0.00g	0.00d	–	–
Hairy vetch (*Vicia villosa*)	0	10.00a	1.58g	0.96e	13.38a	14.87a
	5	6.00b	5.32e	2.26de	7.63b	9.17b
	10	2.50c	10.36d	4.48d	3.90c	4.79c
	15	2.00cd	18.68c	7.35c	2.17d	2.80e
	20	1.75de	21.58b	9.74ab	1.72d	2.37ef
	25	1.00f	24.86a	9.90a	1.52d	2.16f
	30	1.25ef	22.13b	7.33b	1.60d	2.29ef
	35	2.25cd	3.00f	2.00e	3.61c	4.07d
	40	–	0.00h	0.00e	–	–
	45	–	0.00h	0.00e	–	–
Red clover (*Trifolium pratense*)	0	14.25a	0.09g	0.08e	15.63a	16.13a
	5	7.25b	1.23fg	0.73e	14.33a	14.60a
	10	3.25c	3.21ef	1.21cde	6.71b	8.09b
	15	1.50d	8.81d	2.37c	3.41c	4.91c
	20	1.00d	19.92b	4.62b	2.30c	3.33cd
	25	1.00d	23.26a	7.53a	1.60c	2.53d
	30	1.00d	15.05c	3.89b	1.92c	3.09cd
	35	1.25d	4.98e	2.04cd	2.96c	3.70cd
	40	2.00cd	2.34f	0.94de	2.65c	3.44cd
	45	–	0.00g	0.00e	–	–
White clover (*Trifolium repens*)	0	12.25a	0.18d	0.13f	14.63a	15.66a
	5	6.25b	3.10d	1.46f	9.25b	11.27b
	10	3.50c	8.54c	2.92e	3.91d	5.04d
	15	1.50d	20.07b	4.61d	1.70e	2.45e
	20	1.00d	22.79b	7.57b	1.55e	2.11e
	25	1.00d	28.99a	8.95a	1.31e	1.88e
	30	1.00d	30.19a	6.75bc	0.84e	1.76e
	35	1.00d	22.72b	5.79cd	1.04e	2.19e
	40	1.25d	10.10c	2.95e	4.29d	4.64d
	45	3.75c	0.60d	0.31f	7.31c	8.26c
Erect milkvetch (*Astragalus adsurgens*)	0	9.50a	0.33f	0.23ef	13.00a	13.63a
	5	7.00b	2.16e	1.41de	10.99b	11.43b
	10	3.50c	5.51d	2.67cd	5.67c	6.69c
	15	1.25d	14.19b	3.84bc	2.51d	3.48d
	20	1.75d	13.94b	5.97a	2.72d	3.52d
	25	1.25d	16.12a	5.75a	2.00d	2.97d
	30	1.25d	14.20b	6.66a	2.77d	3.43d
	35	2.00d	10.47c	4.30b	2.47d	3.39d
	40	2.00d	4.48d	2.72cd	2.78d	3.40d
	45	–	0.00f	0.00f	–	–
Chinese milkvetch (*Astragalus sinicus*)	0	–	0.00f	0.00f	–	–
	5	11.75a	1.86e	1.36d	16.31a	16.88a
	10	4.25b	4.55d	1.83cd	8.42b	10.91b
	15	2.25cd	8.34b	2.24bc	4.46cd	7.03c
	20	1.00e	14.52a	4.19a	3.73cd	4.68e
	25	1.00e	15.50a	4.74a	3.45d	4.27e
	30	1.75de	6.81c	2.40b	5.56c	6.55cd
	35	2.00cd	3.56d	1.76cd	5.29cd	5.27de
	40	2.75c	1.31ef	0.73e	5.63c	5.91cde
	45	–	0.00f	0.00f	–	–
Niuzhizi (*Lespedeza potaninii*)	0	–	0.00f	0.00d	–	–
	5	18.50a	0.11f	0.10d	19.25a	19.56a
	10	4.75b	2.19e	1.24d	9.69b	10.88b
	15	2.00c	8.35d	3.12c	3.11c	4.14c
	20	2.00c	11.62c	5.72b	2.46c	3.14c
	25	1.00c	22.50a	7.06a	1.30c	2.01c
	30	1.00c	16.27b	5.63b	1.45c	2.15c
	35	1.00c	12.20c	5.20b	1.37c	1.96c
	40	1.00c	11.73c	3.72c	1.64c	2.36c
	45	5.75b	0.70ef	0.41d	10.81b	11.41b
Sweet clover (*Melilotus officinalis*)	0	–	0.00f	0.00g	–	–
	5	6.25a	2.76e	1.30e	11.88a	13.05a
	10	2.75c	6.31d	2.29c	8.44c	9.55c
	15	1.25d	12.06b	3.65b	5.01de	5.97e
	20	1.00d	17.16a	4.60a	3.49e	4.62e
	25	1.00d	15.59a	3.81b	4.91de	5.80e
	30	1.25d	8.72c	1.91d	6.42d	7.63d
	35	3.00c	1.41ef	0.48f	9.00bc	10.16bc
	40	4.50b	0.44f	0.20fg	10.50ab	11.29b
	45	–	0.00f	0.00g	–	–

TSG, time to start germination; GI, germination index; GR, germination rate; T_50_, time taken for 50% germination; MGT, mean germination time. Different lowercase letters within a column and species indicate significant difference at *P* < 0.05 probability level according to Duncan’s multiple range test.

Conversely, GI and GR showed a bell-shaped response (inverse to germination duration parameters), reaching peak values at species-specific optimum temperature ranges (**Table 3**). Notably, germination speed–related parameters such as GR and T_50_ were more sensitive to small temperature fluctuations than GP. For instance, at 10°C, most species achieved relatively high GP values (ranging from 41.0% to 99.0%) (**Figure 1**); however, GR was significantly reduced and T_50_ was markedly delayed compared with values observed at optimum temperature ranges (**Table 3**). Based on germination speed–related parameters, species such as alfalfa and common vetch exhibited relatively faster germination compared with the other forage legume species (**Table 3**).

### 3.3 Germination cardinal temperatures for ten forage legumes

**Figure 2:**
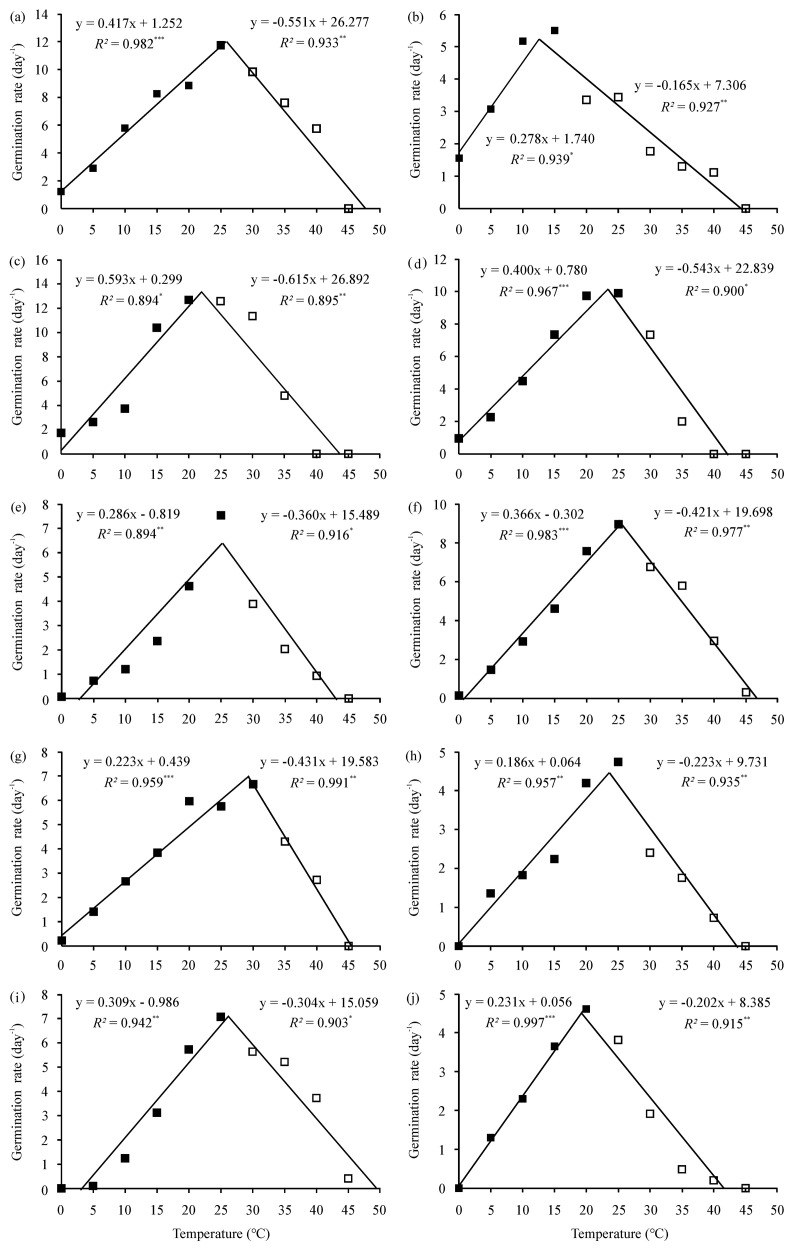
Temperature-dependent germination rate (GR) of ten forage legumes fitted using the intersected-lines model. Symbols represent the observed mean GR at ten constant temperatures, and solid lines represent model predictions. ^*^, ^**^, and ^***^ indicate significance at *P* < 0.05, 0.01, and 0.001 probability level, respectively. **(a)** alfalfa (*Medicago sativa*); **(b)** Yellow medick (*Medicago falcata*); **(c)** common vetch (*Vicia sativa*); **(d)** hairy vetch (*Vicia villosa*); **(e)** red clover (*Trifolium pratense*); **(f)** white clover (*Trifolium repens*); **(g)** erect milkvetch (*Astragalus adsurgens*); **(h)** Chinese milkvetch (*Astragalus sinicus*); **(i)** Niuzhizi (*Lespedeza potaninii*); **(j)** sweet clover (*Melilotus officinalis*).

**Figure 3:**
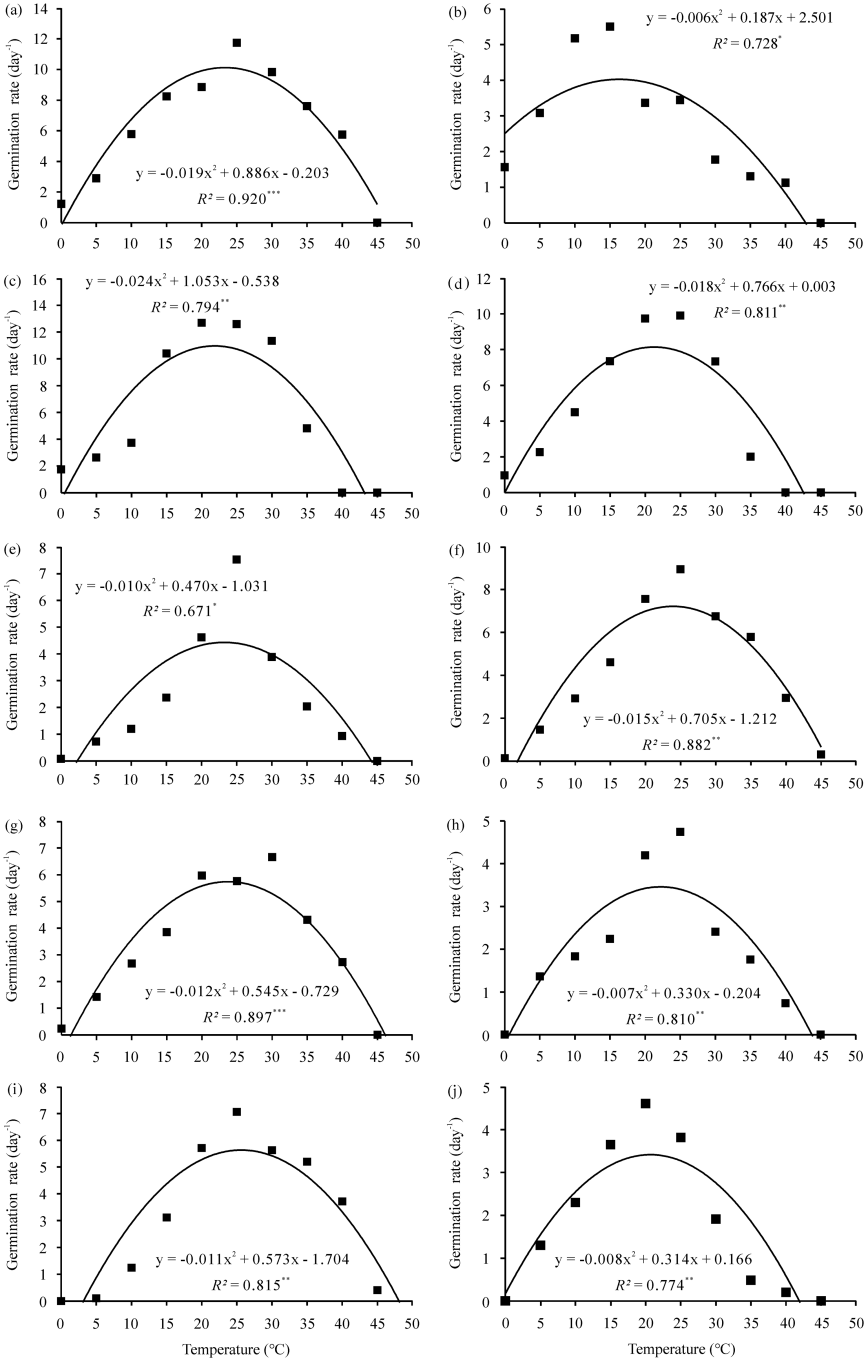
Temperature-dependent germination rate (GR) of ten forage legumes fitted using the quadratic polynomial model. Symbols represent the observed mean GR at ten constant temperatures, and solid lines represent model predictions. ^*^, ^**^, and ^***^ indicate significance at *P* < 0.05, 0.01, and 0.001 probability level, respectively. **(a)** alfalfa (*Medicago sativa*); **(b)** Yellow medick (*Medicago falcata*); **(c)** common vetch (*Vicia sativa*); **(d)** hairy vetch (*Vicia villosa*); **(e)** red clover (*Trifolium pratense*); **(f)** white clover (*Trifolium repens*); **(g)** erect milkvetch (*Astragalus adsurgens*); **(h)** Chinese milkvetch (*Astragalus sinicus*); **(i)** Niuzhizi (*Lespedeza potaninii*); **(j)** sweet clover (*Melilotus officinalis*).

Based on the relationships between temperature and GR described by the two non-linear regression models (**Figures 2**, **3**), germination cardinal temperatures were calculated (**Table 4**). Depending on species and regression model, the estimated base temperature (*T_b_*) for germination ranged from −6.26°C (yellow medick) to 3.19°C (Niuzhizi) using the intersected-lines model, and from −10.22°C (yellow medick) to 3.17°C (Niuzhizi) using the quadratic polynomial model. The estimated optimum temperature (*T_o_*) ranged from 12.57°C (yellow medick) to 29.26°C (erect milkvetch) and from 16.37°C (yellow medick) to 25.56°C (Niuzhizi) based on the intersected-lines and quadratic polynomial models, respectively. In addition, the maximum temperature (*T_m_*) ranged from 41.64°C (sweet clover) to 49.47°C (Niuzhizi) using the intersected-lines model and from 41.85°C (sweet clover) to 47.94°C (Niuzhizi) using the quadratic polynomial model (**Table 4**).

**Table 4:** Germination cardinal temperatures of ten forage legumes based on two non-linear regression models.

Species	Cardinal temperatures (°C)	Intersected-lines	Quadratic polynomial
Alfalfa (*Medicago sativa*)	*T_b_*	– 3.01	0.23
	*T_o_*	25.86	23.31
	*T_m_*	47.67	46.39
	Thermal range (*T_m_*–*T_b_*)	50.68	46.16
	*R^2^*	0.957	0.920
	RMSE	0.795	1.026
Yellow medick (*Medicago falcata*)	*T_b_*	– 6.26	– 10.22
	*T_o_*	12.57	16.37
	*T_m_*	44.28	42.95
	Thermal range (*T_m_*–*T_b_*)	50.54	53.17
	*R^2^*	0.933	0.728
	RMSE	0.443	0.886
Common vetch (*Vicia sativa*)	*T_b_*	– 0.51	0.52
	*T_o_*	22.01	21.85
	*T_m_*	43.71	43.17
	Thermal range (*T_m_*–*T_b_*)	44.22	42.65
	*R^2^*	0.894	0.794
	RMSE	1.618	2.240
Hairy vetch (*Vicia villosa*)	*T_b_*	– 1.95	0.00
	*T_o_*	23.40	21.26
	*T_m_*	42.09	42.53
	Thermal range (*T_m_*–*T_b_*)	44.04	42.53
	*R^2^*	0.933	0.811
	RMSE	0.990	1.610
Red clover (*Trifolium pratense*)	*T_b_*	2.86	2.31
	*T_o_*	25.23	23.29
	*T_m_*	42.99	44.27
	Thermal range (*T_m_*–*T_b_*)	40.13	41.96
	*R^2^*	0.905	0.671
	RMSE	0.783	1.296
White clover (*Trifolium repens*)	*T_b_*	0.83	1.79
	*T_o_*	25.40	23.98
	*T_m_*	46.74	46.17
	Thermal range (*T_m_*–*T_b_*)	45.92	44.38
	*R^2^*	0.980	0.882
	RMSE	0.450	1.004
Erect milkvetch (*Astragalus adsurgens*)	*T_b_*	– 1.97	1.38
	*T_o_*	29.26	23.69
	*T_m_*	45.44	46.00
	Thermal range (*T_m_*–*T_b_*)	47.40	44.62
	*R^2^*	0.975	0.897
	RMSE	0.386	0.720
Chinese milkvetch (*Astragalus sinicus*)	*T_b_*	– 0.34	0.63
	*T_o_*	23.62	22.30
	*T_m_*	43.64	43.98
	Thermal range (*T_m_*–*T_b_*)	43.98	43.35
	*R^2^*	0.946	0.810
	RMSE	0.395	0.657
Niuzhizi (*Lespedeza potaninii*)	*T_b_*	3.19	3.17
	*T_o_*	26.17	25.56
	*T_m_*	49.47	47.94
	Thermal range (*T_m_*–*T_b_*)	46.28	44.77
	*R^2^*	0.922	0.815
	RMSE	0.702	1.081
Sweet clover (*Melilotus officinalis*)	*T_b_*	– 0.24	– 0.52
	*T_o_*	19.25	20.66
	*T_m_*	41.61	41.85
	Thermal range (*T_m_*–*T_b_*)	41.85	42.37
	*R^2^*	0.956	0.774
	RMSE	0.411	0.774

*T_b_*, *T_o_*, and *T_m_* indicate base, optimum, and maximum temperature, respectively. RMSE, root mean square of error.

Based on the intersected-lines and quadratic polynomial models, the widest thermal range for germination was recorded for alfalfa (50.68°C) and yellow medick (53.17°C), respectively (**Table 4**).

Among the two applied models, the intersected-lines model showed better performance in calculating cardinal temperatures and thermal range than the quadratic polynomial model, as indicated by lower RMSE values and higher coefficients of determination (*R²*) across all tested species. Furthermore, based on the intersected-lines model, the highest *R²* among the forage legumes was observed for white clover (0.980), while the lowest RMSE was observed for erect milkvetch (0.386) (**Table 4**).

### 3.4 Correlation between germination cardinal temperatures, thermal range, and related germination parameters

**Figure 4:**
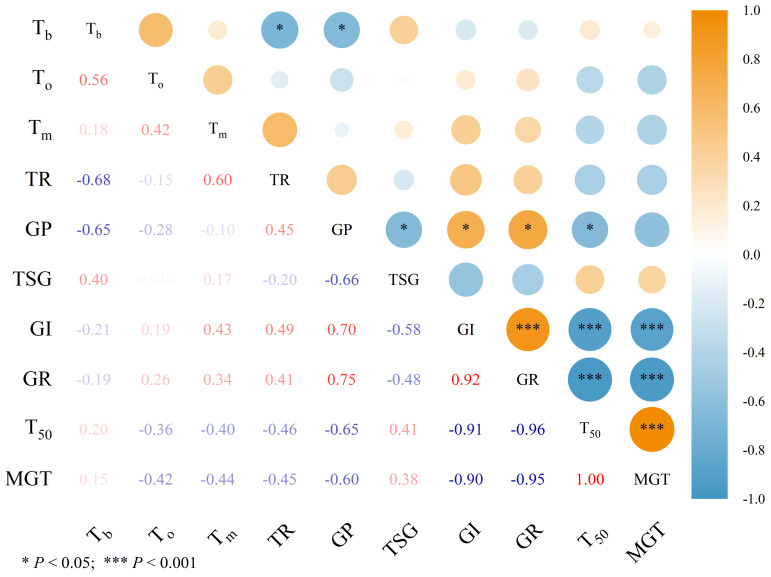
Correlation between germination cardinal temperatures and thermal ranges obtained from intersected-lines model with germination percentage and related germination parameters of ten forage legumes. Germination parameters data used in correlation analysis were average values across all temperature treatments within a species. *T_b_*, base temperature; *T_o_*, optimum temperature; *T_m_*, maximum temperature; TR, thermal range; GP, germination percentage; TSG, time to start germination; GI, germination index; GR, germination rate; T_50_, time taken for 50% germination; MGT, mean germination time. ^*^ and ^***^ indicate significance at *P* < 0.05 and 0.001 probability level, respectively.

**Figure 5:**
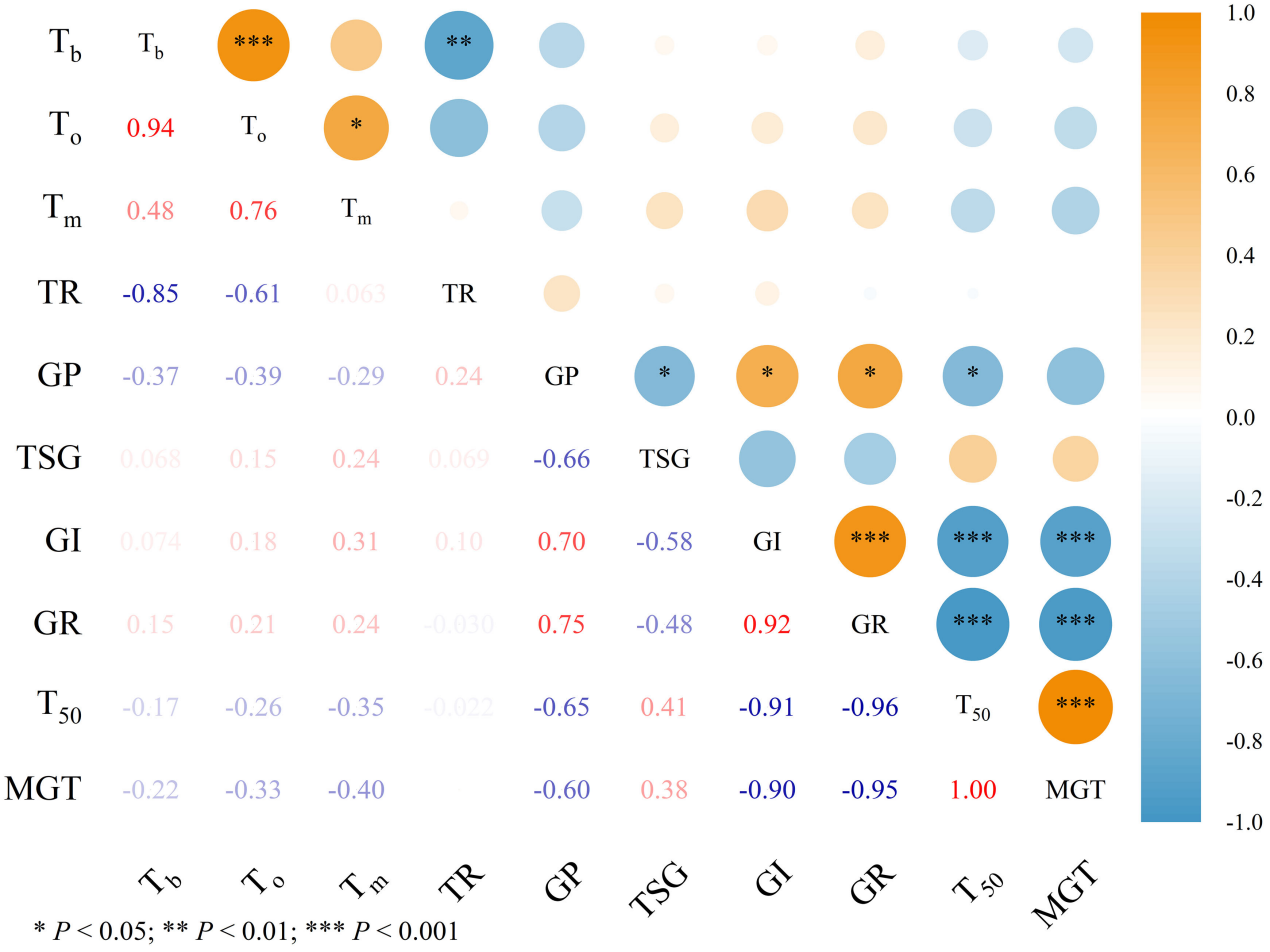
Correlation between germination cardinal temperatures and thermal ranges obtained from quadratic polynomial model with germination percentage and related germination parameters of ten forage legumes. Germination parameters data used in correlation analysis were average values across all temperature treatments within a species. *T_b_*, base temperature; *T_o_*, optimum temperature; *T_m_*, maximum temperature; TR, thermal range; GP, germination percentage; TSG, time to start germination; GI, germination index; GR, germination rate; T_50_, time taken for 50% germination; MGT, mean germination time. ^*^, ^**^ and ^***^ indicate significance at *P* < 0.05, 0.01, and 0.001 probability level, respectively.

To examine the relationships between germination cardinal temperatures, thermal range, and related germination parameters, correlation analysis was conducted (**Figures 4**, **5**). **Figures 4** and **5** illustrate the relationships between cardinal temperatures obtained from the intersected-lines and quadratic polynomial models, respectively, and germination characteristics. Germination percentage (GP) was significantly correlated with all germination parameters except mean germination time (MGT) (*P* < 0.05). Germination index (GI) was significantly positively correlated with germination rate (GR) and significantly negatively correlated with T_50_ and MGT (*P* < 0.001). Germination rate was also significantly negatively correlated with T_50_ and MGT (*P* < 0.001). In addition, a significant positive relationship was observed between T_50_ and MGT (*P* < 0.001). However, no significant correlations were detected between TSG and the other germination parameters.

The base temperature (*T_b_*) was positively correlated with optimum temperature (*T_o_*), particularly when calculated using the quadratic polynomial model (**Figure 5**) (*P* < 0.001). Meanwhile, *T_b_* exhibited a significant negative correlation with the thermal range in both models (**Figures 4**, **5**) (*P* < 0.05). Nevertheless, few significant relationships were detected between germination cardinal temperatures, thermal range, and germination parameters, and no clear relationship was observed between *T_b_* and *T_m_* (**Figures 4**, **5**).

## 4 Discussion

Temperature is widely recognized as a primary regulator of seed dormancy and germination (Hu et al., 2015). Understanding plant thermal requirements through seed germination tests is an effective approach to identifying suitable cultivation areas and sowing windows and, consequently, maximizing yield potential (Tolyat et al., 2014). Forage legumes play important roles in livestock production and environmentally friendly cropping systems; however, their germination, seedling growth, and eventual establishment under field conditions are often constrained by adverse temperature conditions, particularly for small-seeded forage legumes (Wang et al., 2004). Our findings demonstrate that, across all forage legume species examined, all germination parameters were significantly influenced by temperature gradients (**Table 2**). This result is consistent with previous studies, including that of Butler et al. (2014) on warm-season and cool-season forage legumes. Clear differences in germination responses were observed among temperatures and species. From an agronomic perspective, species exhibiting a shorter lag period between seed imbibition and emergence, together with a high germination rate (GR), may be particularly suitable for early sowing to promote seedling establishment well before the onset of summer drought and heat stress (Zhang et al., 2013). Notably, two annual forage legumes (common vetch and hairy vetch) in the present study showed relatively higher GR compared with most perennial or biennial species, except for alfalfa (**Table 3**). This observation is consistent with the findings of Maleki et al. (2024), who reported that annual crops generally germinate more rapidly than wild species. As a perennial forage legume, alfalfa also exhibited rapid germination, which may reflect the effects of domestication, given its long history as a globally important forage crop.

Germination percentage (GP), germination index (GI), and GR were low, whereas time to start germination (TSG), time taken for 50% germination (T_50_), and mean germination time (MGT) were high under both low and high temperature regimes (**Figure 1**; **Table 3**). These responses likely result from reduced enzyme activity and metabolic rates at low temperatures or from the induction of secondary dormancy at high temperatures (Cave et al., 2011). The decline in GP, GI, and GR at sub-optimal temperatures may be associated with reduced metabolic activity and impaired enzyme kinetics, such as those of α-amylase, which plays a key role in starch mobilization in legumes (Moot et al., 2000). Conversely, supra-optimal temperatures approaching *T_m_* may lead to thermo-inhibition or secondary dormancy in forage legumes, often involving membrane destabilization and the synthesis of heat-shock proteins (Teixeira et al., 2020). Previous studies have reported similar temperature-dependent germination responses, including faster germination of white clover at 15–25°C (Chu et al., 2022), hairy vetch (Yang et al., 2022), and sweet clover (Ghaderi-Far et al., 2010). In addition, germination speed–related parameters appeared more sensitive to temperature variation than GP. For example, the decoupling of GP and GR observed at 10°C suggests that low temperature primarily limits the kinetic energy of biochemical reactions rather than seed viability per se (Moot et al., 2000).

It should also be noted that the present study employed constant temperature regimes to quantify germination cardinal temperatures and thermal ranges, whereas soil temperatures under field conditions fluctuate diurnally. In many species, alternating temperatures have been shown to enhance germination compared with constant temperatures (Lin et al., 2017; Kallow et al., 2020), potentially by alleviating physical dormancy or facilitating metabolic repair during cooler phases (Van Assche et al., 2003). However, other studies have reported no significant differences in GP or GR between constant and alternating temperature regimes, indicating that some species do not require temperature alternation for germination (Ortega-Baes et al., 2011; Yang et al., 2022). Masin et al. (2017) suggested that, for species exhibiting low germination under constant temperatures, further testing using alternating temperature regimes may be necessary to reliably estimate cardinal temperatures. In the present study, germination cardinal temperatures were determined for ten forage legumes, and both GP and GR were high under optimal temperature ranges. From a modeling perspective, these results indicate that cardinal temperatures derived from constant temperature regimes remain a robust and fundamental basis for predicting germination responses. Indeed, constant-temperature approaches have been widely applied to estimate cardinal temperatures and seed vigor across numerous plant species (Hardegree, 2006; Fallahi et al., 2017; Zhang et al., 2020; Solouki et al., 2022), consistent with the methodology used here.

The application of mathematical models provides a powerful means of quantifying the effects of environmental factors on germination characteristics across diverse species (Bakhshandeh et al., 2020; Zhang et al., 2022). To date, several models—including intersected-lines, segmented, beta, dent, and quadratic polynomial models—have been successfully applied to describe temperature-dependent germination responses (Bidgoly et al., 2018; Cabrera-Santos et al., 2022). Parameters estimated using these models can serve as critical inputs for predicting seed germination and seedling emergence under field conditions and future climate scenarios. Chen et al. (2021) emphasized that GR is closely related to temperature, supporting the development of GR-based models to describe progress toward germination. It is well established that GR increases linearly with temperature from *T_b_* to *T_o_* and subsequently declines toward zero at supra-optimal temperatures (Kebreab and Murdoch, 1999; Parmoon et al., 2015). This pattern was confirmed in the present study, as GR and related parameters increased with temperature up to *T_o_* and then decreased beyond this threshold (**Table 3**). In this study, two commonly used non-linear regression models—the intersected-lines and quadratic polynomial models—were applied to estimate germination cardinal temperatures for ten forage legumes. Both models demonstrated satisfactory accuracy based on RMSE and *R²* values; however, the intersected-lines model consistently outperformed the quadratic polynomial model (**Table 4**). This finding aligns with previous work by Fallahi et al. (2017), who also identified the intersected-lines model as the most suitable for describing temperature-dependent germination responses in flixweed (*Descurainia sophia*).

It is noteworthy that germination cardinal temperatures and thermal range varied among the different forage legumes studied (**Table 4**). Germination characteristics are influenced not only by environmental factors but also differ among species and even among cultivars (Ali et al., 1994). This variation is particularly important for understanding plant growth potential across diverse geographic regions and, consequently, variability in crop yields (Massawe et al., 2003). For example, for the two species from the genus *Vicia* examined in this study, the calculated *T_o_* ranges are consistent with previous findings by Mosjidis and Zhang (1995), who reported 18–23°C as the optimal temperature range for germination across 15 accessions of six *Vicia* species. Based on both models, the highest *T_b_* and *T_m_* values were observed for Niuzhizi, indicating a warm-temperature adaptation during the germination stage for this species. Niuzhizi is a native legume endemic to arid regions of northwest China and plays important roles in local desert restoration (Chen et al., 2024). Its enhanced tolerance to high temperatures during germination likely contributes to its suitability for ecological restoration under harsh environmental conditions. The *T_b_* of alfalfa obtained in the present study deviated slightly from the value of 2.9°C reported by Sakanoue (2010) and from the range of −0.55 to 0.49°C reported across cultivars by Jungers et al. (2016). These differences are likely attributable to variation among cultivars used in the assays and may also reflect differences in modeling approaches (e.g., the use of GR versus 1/T_50_). Notably, *T_o_*, *T_m_*, and thermal range values were not reported in the aforementioned studies. The two *Medicago* species examined here also exhibited wider germination thermal ranges compared with the other forage legumes (**Table 4**), suggesting broader environmental adaptation. This finding is consistent with the well-established global importance and wide distribution of species within the genus *Medicago* (Zhong et al., 2025). From an agronomic perspective, quantifying germination cardinal temperatures provides a precise framework for optimizing sowing windows. For instance, knowledge of *T_b_* allows agronomists and growers to determine the earliest safe sowing date in spring to avoid frost-induced seedling mortality, whereas *T_m_* helps to avoid exposure to late-summer heat stress during establishment (Moot et al., 2000). Interestingly, several species—particularly within the genus *Medicago*—exhibited *T_b_* values below 0°C. Although these negative values result from mathematical extrapolation of non-linear regression models, they may carry important biological and ecological implications. A *T_b_* < 0°C suggests a high degree of cold tolerance and an “early-emergence” strategy, allowing seeds to initiate metabolic activity immediately following spring thaw. Biologically, the presence of cryoprotective compounds within seed embryos may permit biochemical reactions at sub-zero temperatures while minimizing cellular damage. Species exhibiting *T_b_* < 0 °C therefore possess a wider sowing window, facilitating earlier establishment and improved utilization of spring soil moisture. Sub-zero *T_b_* values have also been reported for other plant species, including forage brassicas (*Brassica napus*) (Andreucci et al., 2016), *Saxifraga tridactylites* (Trudgill et al., 2000), barley (*Hordeum vulgare*) (Zhang et al., 2010), and *Cryptantha minima* (Wei et al., 2009). Furthermore, identifying species-specific thermal niches enables more accurate matching of forage legumes to regional climatic conditions, thereby enhancing the reliability and sustainability of legume-inclusive cropping systems (Donohue et al., 2010; Ditzler et al., 2021).

Beyond immediate sowing optimization, germination cardinal temperatures—particularly *T_o_* and *T_m_*—are critical for assessing species resilience under climate change. According to the framework proposed by Sentinella et al. (2020), a species’ vulnerability to warming is determined not only by the breadth of its thermal tolerance but also by the proximity of its upper thermal limit (*T_m_*) to current and projected environmental temperature extremes. Species with relatively low *T_m_* values identified in this study, such as sweet clover, may therefore face increased risk under future warming scenarios, as germination temperatures may approach or exceed their upper thermal thresholds. This risk may be particularly pronounced in high northern latitude regions, which are projected to experience greater warming than tropical and southern temperate regions (Sentinella et al., 2020).

Results from correlation analysis revealed no statistically significant relationships between cardinal temperatures and germination parameters (**Figures 4**, **5**), suggesting that, within a species, these cardinal thresholds are relatively stable traits that may vary independently of overall germination vigor. However, whether the cardinal temperatures of the ten forage legumes examined here are influenced by other environmental factors, such as water potential and salinity stress, requires further investigation. Significant relationships between *T_b_* and *T_o_*, as well as between *T_b_* and thermal range, were observed in this study. These findings are consistent with the global database updated by Maleki et al. (2024), suggesting that plants adjust *T_b_* in coordination with other cardinal temperatures as an efficient adaptive strategy for coping with unfavorable environmental conditions. The significant correlations observed among several germination parameters across the ten tested species are consistent with previous reports and are not unexpected (Khan et al., 2023; Lv et al., 2024).

## 5 Conclusion

The present experiment quantified, for the first time, the germination cardinal temperatures (*T_b_*, *T_o_*, and *T_m_*) and thermal ranges for several important forage legumes, providing a valuable supplement to the existing global seed trait database. Temperature exerted a strong influence on the seed germination characteristics of the ten forage legumes studied. Germination percentage (GP), germination index (GI), and germination rate (GR) increased and then declined with increasing temperature, whereas time to start germination (TSG), time taken for 50% germination (T_50_), and mean germination time (MGT) showed the opposite trend. In general, all species exhibited relatively high GP and rapid germination at temperatures between 15 and 25°C. Both non-linear regression models applied in this study adequately described germination responses to temperature across the ten species, although the intersected-lines model consistently outperformed the quadratic polynomial model. Based on these models, germination cardinal temperatures and thermal ranges varied markedly among the forage legumes, allowing their categorization according to thermal preferences. Species such as alfalfa, yellow medick, and erect milkvetch exhibited broad germination thermal ranges, characterized by low *T_b_* values, high *T_m_* values, and wide thermal tolerance, enabling effective germination across diverse geographic regions and seasons, provided that other environmental conditions are favorable. Sweet clover favored cooler temperature regimes for germination, as evidenced by its comparatively low *T_o_* and *T_m_* values. In contrast, Niuzhizi and white clover favored higher temperature conditions, reflected by their higher *T_o_* and *T_m_* thresholds. Species within the genus *Vicia* and red clover were better adapted to moderate temperature regimes. No statistically significant relationships were detected between germination cardinal temperatures and germination parameters, indicating that germination cardinal temperatures primarily reflect the degree of environmental adaptation of a species rather than its overall germination vigor. Knowledge of germination cardinal temperatures is therefore essential for informed decision-making regarding geographic region selection, sowing practices, and efficient forage legume production. Moreover, these data contribute to a deeper understanding of species distribution patterns in response to climate change. Future research should focus on integrating additional environmental factors—such as water potential and saline–alkaline stress—into germination modeling frameworks.

## Data Availability

The original contributions presented in the study are included in the article/**Supplementary Material**. Further inquiries can be directed to the corresponding authors.
